# Effects of the COVID-19 Pandemic on the Frequency of Bystander Intervention in Out-of-Hospital Cardiac Arrests

**DOI:** 10.7759/cureus.50353

**Published:** 2023-12-11

**Authors:** Simon P Alarcon, Jordan L Pace, James McDermott, Sam MacDowell, Shazia Sheikh

**Affiliations:** 1 Medicine, California University of Science and Medicine, Colton, USA; 2 Medical Education, California University of Science and Medicine, Colton, USA

**Keywords:** pandemic, arrest, cardiac, bystander, cpr, covid

## Abstract

Aim: Rapid administration of cardiopulmonary resuscitation (CPR) can significantly increase patient survival following an out-of-hospital cardiac arrest (OHCA). Through this study, we aimed to determine if the onset of the coronavirus disease 2019 (COVID-19) pandemic affected the likelihood of OHCA victims receiving bystander-initiated CPR prior to EMS arrival.

Methods: We used data collected by the National Emergency Medical Services Information System (NEMSIS) for years 2019 and 2020. Data was filtered to include only cases of OHCA where the status of bystander CPR was listed. We used a chi-square analysis to compare frequencies of patients receiving both bystander CPR and standard EMS interventions versus patients receiving only standard EMS interventions for the years before and during the COVID-19 pandemic declaration (2019 and 2020, respectively).

Results: Of the 577,011 cases that met our inclusion criteria, 228,259 occurred in 2019 and 348,752 occurred in 2020. The frequency of OHCA cases that reported bystander-initiated CPR prior to EMS arrival significantly decreased from 2019 to 2020 (53.7% vs. 52.5%, P<.001).

Conclusion: Bystanders are often the first to administer CPR following a cardiac arrest. It was found that the likelihood of an OHCA victim receiving bystander CPR decreased from 2019 to 2020.

## Introduction

Annually, over 356,000 cardiac arrests occur in the United States outside of a hospital setting [1]. Following an out-of-hospital cardiac arrest (OHCA), rapid administration of cardiopulmonary resuscitation (CPR) can significantly increase patient survival. Data reported in 2019 found that adults suffering an OHCA who received CPR from a layperson demonstrated a reduction in hospital mortality as well as an increase in the probability of survival to discharge, and good neurologic outcomes on discharge compared to adult OHCA victims who did not receive CPR from a layperson [2].

The onset of the coronavirus disease 2019 (COVID-19) pandemic marked an increased incidence of OHCAs. These effects were likely due to direct effects of COVID-19 infection as well as delayed access to healthcare and reluctance to seek medical attention for the fear of increased COVID-19 exposure [1,3,4]. Despite the increase in OHCAs, a survey performed by Grunau et al. indicated a decreased willingness to perform CPR in 2020 compared to prior years [5,6]. Bystander CPR, which here signifies CPR administered prior to the arrival of the EMS, may be discouraged by a reduction in CPR training, or risk of exposure to potentially infectious respiratory secretions [2,5]. To date, several studies have outlined changes in CPR characteristics and public willingness to perform CPR following the COVID-19 outbreak in the United States [2,4,6]. To our knowledge, no studies have assessed changes in the frequency of bystander CPR at a national level for greater than six months [2,4,6,7]. Our study seeks to determine if the frequency of bystander-initiated CPR encounters in the United States was significantly reduced by the onset of the COVID-19 pandemic.

## Materials and methods

Study design

A retrospective observational study design was implemented to evaluate the impact of the COVID-19 pandemic on the frequency of bystander-initiated CPR provided to OHCA victims before the arrival of EMS.

Data source and description

The National Emergency Medical Services Information System (NEMSIS) database was utilized in our study. The NEMSIS database is a federally funded national repository that collates standardized EMS activation details from over 10,000 EMS agencies throughout the United States. It offers a comprehensive perspective on pre-hospital care trends and practices.

Data collection protocols

Our team accessed data from the NEMSIS database for two calendar years: 2019 (pre-COVID-19) and 2020 (post-COVID-19) [7,8]. We followed all necessary protocols and secured approvals from the California University of Science and Medicine (CUSM) institutional review board to ensure ethical standards were met during data extraction and analysis.

Participant selection

Inclusion/Exclusion Criteria

**Figure 1:**
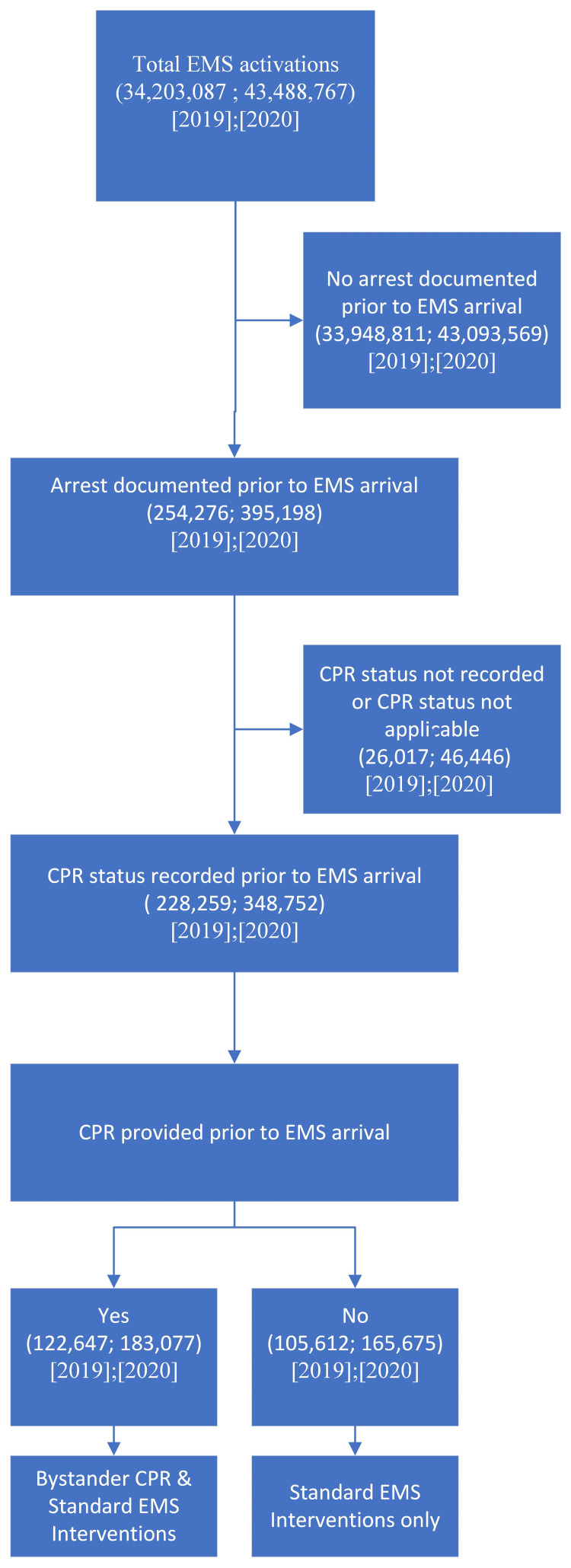
NEMSIS selection criteria NEMSIS, National Emergency Medical Services Information System; CPR, cardiopulmonary resuscitation

We established specific criteria to filter relevant cases for our analysis as outlined in Figure 1. The primary focus was on EMS activations where the primary medical emergency was identified as an OHCA and information about CPR administration prior to EMS arrival (bystander CPR) was explicitly documented. Cases with missing data or where the status of CPR administration before EMS arrival was ambiguous were excluded from the analysis.

Dataset definition

The data from 2019 served as our control group, representing the period before the declaration of the COVID-19 pandemic. In contrast, the 2020 data acted as our study group, reflecting the period after the pandemic's declaration. Calendar years were selected as the defining bounds of the COVID-19 pandemic to capture as many possible instances where the administration of bystander CPR could have been impacted by the COVID-19 pandemic. January 2020 was used as the starting month of the COVID-19 pandemic for our study for several reasons. First, this was the month where COVID-19 was initially identified in the United States [9]. Second, previous research on news articles suggests widespread media coverage of the COVID-19 pandemic prior to the lockdowns that occurred in March of 2020 [10]. Finally, we used the month of January as the starting month because this is the month when the Secretary of the Department of Health and Human Services of the United States declared the 2019 novel coronavirus outbreak a public health emergency [11].

Statistical analysis

All statistical computations were performed using the SPSS Statistics software suite (IBM Corp., Armonk, NY), known for its robust data-processing capabilities for biomedical research. We employed a chi-square test, which is suitable for analyzing categorical data and determining if significant differences exist between expected and observed frequencies in one or more categories. Specifically, we compared the observed frequencies of OHCA patients in 2019 and 2020 who received both bystander CPR and standard EMS interventions versus those who received only standard EMS interventions. To quantify the likelihood of an event occurring in one group compared to another, we calculated the odds ratio. In our study, this meant determining the odds that CPR would be initiated by a bystander in 2020 compared to 2019. An odds ratio of 1 implies that the event is equally likely in both groups, above 1 indicates an increased likelihood, and below 1 denotes a decreased likelihood.

## Results

A total of 577,011 cases from the NEMSIS database met study inclusion criteria. In 2019, 53.7% of patients (122,647 patients) received bystander CPR in addition to standard EMS interventions, compared to 46.3% of patients (105,612 patients) who received standard EMS interventions only (Table 1). In 2020, 52.5% (183,077 patients) received bystander CPR and standard EMS interventions, compared to 47.5% (165,675 patients) who received standard EMS interventions only. Therefore, there was a 1.2% decrease in the frequency of OHCA patients receiving bystander CPR in 2020 relative to 2019, with an odds ratio of 0.952 times chance that a victim would receive bystander CPR in 2020 compared to 2019 (Table 2).

**Table 1:** CPR administration provider frequency counts and percentages for years 2019 and 2020 CPR, cardiopulmonary resuscitation; EMS, Emergency Medical Services

CPR status	Frequency counts	2020 (during COVID-19)	2019 (pre-COVID-19)	Total
Bystander CPR and standard EMS interventions	Observed count (percent)	183077 (52.5)	122647 (53.7)	305,724
	Expected count (percent)	184783.1 (52.98)	120940.9 (52.98)	305,724
Standard EMS interventions only	Observed count (percent)	165675 (47.5)	105612 (46.3)	271,287
	Expected count (percent)	163968.9 (47.02)	107318.1 (47.02)	271,287
Total	Observed count (percent)	348752 (100)	228259 (100)	577,011
	Expected count (percent)	348752 (100)	228259 (100)	577,011

**Table 2:** Calculated odds ratio CPR, cardiopulmonary resuscitation

Statistical analysis	Value	Lower 95% CI	Upper 95% CI
Odds ratio (bystander CPR after the pandemic/bystander CPR before the pandemic)	0.952	0.942	0.962

Pearson chi-square value, continuity correction, and likelihood ratio for cardiac arrests with CPR administered by bystanders and standard EMS interventions versus standard EMS interventions only were also calculated. The Pearson chi-square analysis yielded a value of 84.691, continuity correction yielded a value of 84.641, and the likelihood ratio test revealed a value of 84.711, each with a two-sided asymptotic significance of <0.001 (Table 3).

**Table 3:** Calculated chi-square results

Statistical analysis	Value	df	Asymptotic significance (two-sided)
Pearson chi-square	84.691	1	<0.001
Continuity correction	84.641	1	<0.001
Likelihood ratio	84.711	1	<0.001

## Discussion

We hypothesized that the COVID-19 pandemic was associated with a significant decrease in the frequency of bystander CPR. The Pearson chi-square test, with an asymptotic significance of <0.001, allows for the rejection of the null hypothesis, and acceptance that there is a statistically significant difference in the frequency of bystander CPR administration between the years 2019 and 2020. This is further supported by the data from the continuity correction and likelihood ratio tests. The results therefore suggest that CPR administration by bystanders decreased during the COVID-19 pandemic. Furthermore, the odds ratio indicates that there was 0.952 times chance that CPR would be initiated by bystanders in 2020 compared to 2019. This suggests that there was a small reduction in the likelihood of CPR being performed by bystanders during the COVID-19 pandemic.

Identifying decreases in bystander-administered CPR during the COVID-19 pandemic is integral to understanding potential reasons for this behavior change and finding solutions to combat it. Detecting declines in bystander-performed CPR can help public health programs create strategies to ensure the public feels comfortable administering CPR in out-of-hospital cardiac arrest cases. A previous research by Chan et al. demonstrated a decrease in rates of return of spontaneous circulation (ROSC) in OCHA during the COVID-19 pandemic and this finding correlates with the outcome of our study [12]. A decline in ROSC could be partially explained by a decrease in bystander-performed CPR. Increased bystander-performed CPR is known to lead to increased cases of ROSC in OHCA [13]. Therefore, a decrease in bystander-performed CPR as shown by our research could partially explain the observed decrease in return of spontaneous circulation that occurred during the COVID-19 pandemic. Our research further expands upon the previous research by Shekhar et al. that looked at the first six months of the COVID-19 pandemic and established declines in bystander-performed CPR through monthly comparisons [14]. Our research expands upon their work by extending the time period of analysis and calculating more comparative statistics to better evaluate for potential pre- and post-COVID-19 differences.

While our study focuses on OHCA, studies have shown that decreasing rates of CPR during the pandemic also negatively impacted adults with in-hospital cardiac arrest (IHCA) [15]. Additionally, research has shown that patients with active COVID-19 infection who developed IHCA and consequently received CPR had poor outcomes. In a retrospective study of patients with COVID-19 who suffered from IHCA and received CPR, only 13.2% achieved ROSC and only 2.9% survived beyond 30 days [16]. Poor outcomes among COVID-19 patients who received IHCA CPR would imply that OHCA bystander CPR would have less of an effect on patients who were infected with COVID-19. Similarly, a cross-sectional study demonstrated a ROSC of 9% and only 2% surviving to discharge [17]. In a case series of 54 patients, zero survived to discharge [18]. Further research may explore whether the consequences of decreased CPR administration had negative outcomes for both patients with and without COVID-19 infection, or if patients without COVID-19 infection were disproportionately affected considering COVID-19 patients may have reduced survival regardless of CPR administration. In a study including only patients without COVID-19 infection, Tong et al. found that fewer patients received CPR by first-responders and that arrival to the scene was delayed, both leading to decreased ROSC [19]. While our focus is on the overall impact of COVID-19 on the administration of CPR for OHCA, the consequences are important to consider in all settings and all patients.

Limitations

While the results of this study demonstrate a significant reduction in the frequency of bystander CPR performed during the COVID-19 pandemic, the study design and choice of database limit the ability to make further interpretations.

First, because COVID-19 lockdowns did not occur until March of 2020, it is likely that many of the negative public health effects of the pandemic were not felt during the early months of 2020. Defining pre- and post-pandemic years as 2019 and 2020, respectively, likely reduced our calculated odds ratio because fewer lay people were probably aware of the COVID-19 cases that were occurring during this time.

Second, our retrospective observational study cannot single out the COVID-19 pandemic as the causal factor in the decline. Appreciating how regional and temporal differences of the COVID-19 pandemic affect bystander CPR would help to establish a causal link between the COVID-19 pandemic and the decrease in bystander-initiated CPR and may help to elucidate specific aspects of the pandemic that led to the decline. Additionally, while the NEMSIS database is one of the most comprehensive tools available to evaluate overall trends in prehospital care, EMS agencies are not required to submit patient cases to the database. As such, the database is a convenience sample and does not necessarily reflect more detailed regional or temporal differences in bystander-administered CPR.

Third, the NEMSIS database fails to capture and isolate tiered-level EMS responses. Therefore, there is likely an overcount of the number of cardiac arrest cases reported by the NEMSIS database. The inability to account for tiered EMS responses should however not affect the outcome of this study because it is unlikely that tiered responses occur in a biased fashion with respect to the bystander CPR status.

## Conclusions

In conclusion, bystanders are often the first to administer CPR following a cardiac arrest. The onset of the COVID-19 pandemic was associated with a small but significant decrease in the frequency of cardiac arrest victims who received bystander CPR prior to EMS arrival. Further examination of factors driving this change may help establish useful targets for public messaging and education regarding CPR administration.
